# Pharmacokinetics and drug delivery systems for puerarin, a bioactive flavone from traditional Chinese medicine

**DOI:** 10.1080/10717544.2019.1660732

**Published:** 2019-09-14

**Authors:** Liang Zhang

**Affiliations:** College of Animal Pharmaceutical Sciences, Jiangsu Agri-animal Husbandry Vocational College, Taizhou, PR China

**Keywords:** Puerarin, poorly soluble drug, pharmacokinetics, metabolites, drug delivery systems, microemulsions, dendrimers, nanoparticles, nanocrystals

## Abstract

*Pueraria lobata* (Willd.) Ohwi is a medicinal and edible homologous plant with a long history in China. Puerarin, the main component isolated from the root of *Pueraria lobata*, possesses a wide range of pharmacological properties. Daidzein and glucuronides are the main metabolites of puerarin and are excreted in the urine and feces. As active substrates of P-gp, multidrug resistance-associated protein and multiple metabolic enzymes, the pharmacokinetics of puerarin can be influenced by different pathological conditions and drug-drug interactions. Due to the poor water-solubility and liposolubility, the applications of puerarin are limited. So far, only puerarin injections and eye drops are on the market. Recent years, researches on improving the bioavailability of puerarin are developing rapidly, various nanotechnologies and preparation technologies including microemulsions and SMEDDS, dendrimers, nanoparticles and nanocrystals have been researched to improve the bioavailability of puerarin. In order to achieve biocompatibility and desired activity, more effective quality evaluations of nanocarriers are required. In this review, we summarize the pharmacokinetics and drug delivery systems of puerarin up to date.

## 1. Introduction

Puerarin is the major bioactive ingredient isolated from the root of *Pueraria lobata* (Willd.) Ohwi, which is well known as Gegen in traditional Chinese medicine. Gegen is a medicinal and edible homologous plant with a long history in China. Puerarin was isolated from Gegen in the late 1950s (Zhang et al., 2019). Since then, the pharmacological effects of puerarin have been extensively investigated. Puerarin possesses a wide range of pharmacological properties, which has been widely used in the treatment of cardiovascular and cerebrovascular diseases, diabetes and diabetic complications, osteonecrosis, Parkinson's disease, Alzheimer's disease, endometriosis, and cancer (Zhou et al., 2014). However, despite possessing broad pharmacological activities, the clinical applications of puerarin are limited. According to the database of China Food and Drug Administration (http://app1.sfda.gov.cn/datasearchcnda/face3/dir.html), intravenous injections and eye drops seem to be the only drug delivery methods of puerarin.

**Figure 1.**
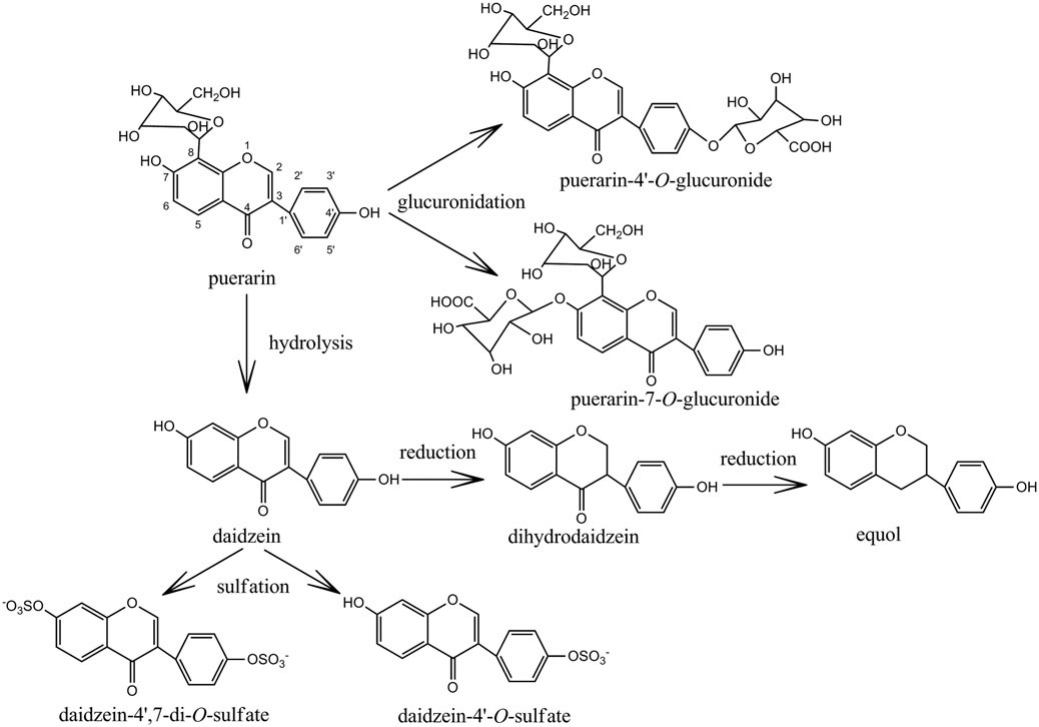
Chemical structures of puerarin and its metabolites.

Puerarin (7,4’-dihydroxy-8-C-glucosylisoflavone, Figure 1) belongs to the chemical structure of isoflavones. B ring is influenced by the stereo-hindrance of carbonyl group of pyran ring, which forms a large conjugate system to be an approximate planar structure in space. Besides, two phenolic hydroxyl groups at the 7,4’-site can form intermolecular hydrogen bonds, and which could increase the intermolecular force and melting point of puerarin (Lv & Tan, 2009). The chemical structural characteristics of puerarin contribute to the poor water-solubility and liposolubility, thereby leading to poor oral absorption and low bioavailability, which eventually restricts its wide application in clinic.

Puerarin could be categorized IV drug of the biopharmaceutics classification system based on its low solubility and low intestinal permeability values, with an aqueous solubility of 0.46 mg/mL and a maximum solubility of 7.56 mg/mL at pH 7.4 in phosphate buffers (Quan et al., 2007; Li et al., 2014). To improve solubility of puerarin, cosolvents such as propylene glycol, ethylene glycol and polyvinylpyrrolidone had been added to the clinical injection formulation. However, adverse drug reactions caused by cosolvents after intravenous administration such as vascular stimulation, fever, allergy and erythrolysis increased year by year (Deng & Zhang, 2008). Besides, due to the short elimination half-life, administer frequent or high doses of injection is needed. Thus, a new drug delivery system for puerarin has important significance. Oral formulations with improved absorption of puerarin have attracted widespread attention. Recent years, a series of researches on improving oral bioavailability are reported, strategies include nanotechnologies and various preparation technologies.

Rational drug therapy is dependent upon a basic understanding of the way that patients handle drugs (pharmacokinetics) and their response to specific drug effects (pharmacodynamics). Since knowledge of the pharmacokinetic processes could help us to explain and predict a variety of events related to the efficacy and toxicity of herbal preparations, it is important to do some pharmacokinetics investigations of puerarin for further evaluation of its clinical applications. Besides, understanding the metabolic pathways of puerarin will contribute to the understanding of its therapeutic as well as toxic effects.

In this article, pharmacokinetics and new drug delivery systems for puerarin are reviewed up to date.

## 2. Pharmacokinetics of puerarin

Species specificity was found in pharmacokinetics of puerarin. The pharmacokinetics of puerarin in plasma of rats and dogs coincides with the two-compartment open model (Jin & Zhu, 1992; Yang et al., 2011; Ouyang et al., 2012), while in rabbits was best fitted to the three-compartment open model (Jin & Zhu, 1992).

After intravenous injection, puerarin was detected in most organs, including the hippocampus, heart, lung, stomach, liver, mammary gland, kidney, spleen, tibia, and femur (Anukunwithaya et al., 2018). Puerarin could cross the blood-brain barrier, then widely distribute in areas of the brain such as hippocampus, cerebral cortex, and the striatum (Kong et al., 2017). The excretion of puerarin into the bile in the form of unconjugated puerarin was also detected (Prasain et al., 2009). Puerarin could penetrate the placental barrier and maintain high concentrations in fetal rat plasma, which indicated puerarin administration should be carefully in pregnant woman (Cao et al., 2013).

A few phase I functionalization reaction (reduction and hydrolysis) and phase II conjugation reaction (glucuronidation and sulfation) metabolites have been found in biological samples after puerarin administration to both humans and animals (Figure 1). The major hydrolysis metabolite of puerarin was identified as daidzein (Prasain et al., 2004; Wen et al., 2008; Jung et al., 2014), which was formed by CYP 450 (cytochrome P450 proteins) in the liver microsomes (Wen et al., 2008), and subsequently reduced to dihydrodaidzein and equol (Prasain et al., 2004). Daidzein could also form daidzein-4’,7-di-*O*-sulfate and daidzein-4’-*O*-sulfate through the sulfation progress (Yasuda et al., 1995). Glucuronides were the main metabolites of puerarin and were excreted in the urine and feces (Anukunwithaya et al., 2018). The glucuronides conjugates of puerarin such as puerarin-7-*O*-glucuronide and puerarin-4'-*O*-glucuronide were the main metabolites of conjugation reaction (Luo et al., 2010, 2012; Anukunwithaya et al., 2018). Seven UDP-glucuronosyl-transferase (UGT) isoforms, including UGT1A1, 1A9, 1A10, 1A3, 1A6, 1A7 and 1A8, could catalyzed the formation of puerarin-7-*O*-glucuronide, and UGT1A1 was the primary enzyme responsible for puerarin metabolism in human liver microsomes (Luo et al., 2012). The metabolic profiles were similar in rat liver and intestine, indicating that no metabolic regioselectivity of puerarin occurs in rat liver and intestine (Luo et al., 2010).

Besides, the metabolic profiles of puerarin seemed to differ from tissues and biological samples. The deconjugated and reductive metabolites daidzein, dihydrodaidzein, and equol were detected in 4 h rat urine samples following oral administration (Prasain et al., 2004). However, unmetabolized puerarin was detected in the serum samples collected at 4 h, and only equol was also detected in very low quantity until in the 24 h blood sample, which suggested that puerarin was rapidly absorbed from the intestine without metabolism.

### 2.1. Effects of pathological conditions on pharmacokinetics of puerarin

Many pathological conditions could potentially affect the pharmacokinetics of drugs. The changes of pharmacokinetic parameters in different disease states could guide the use of puerarin in clinical application. Puerarin has been proved to be effective on Type II diabetes mellitus (Dong et al., 2018). Under intravenous and oral administration routes, pharmacokinetic parameters such as area under the curve(AUC), mean residence time(MRT), and clearance (CL) in the diabetes mellitus rats statistically differed from those in the control rats. For the oral route, the AUC, C_max_, and half time (t_1/2_) of the analytes were significantly lowered in the diabetes mellitus rats, whereas the clearance divided by the absorption fraction and the apparent volume of distribution divided by the absorption fraction values were significantly higher in the control rats. The differences were greater with oral dosing than with intravenous dosing, which we should take regard in clinical application. Meanwhile, the hepatic and intestinal gene and protein expressions of Ugt1a1 and Ugt1a7 were significantly increased in the diabetes mellitus rats.

Puerarin is likely to be a substrate of P-gp, which was reported up-regulated in liver and intestines of many liver disease animal models (Liang et al., 2012; Liao et al., 2014). Puerarin concentrations of the hepatic fibrosis group were significantly lower than those of the corresponding control group at almost any time after 5 mg/kg or 20 mg/kg intravenous administrated, which could attribute to the increased expression of P-gp, Ugt1a1, and Ugt1a7 in liver and intestines of hepatic fibrosis rats (Zhang et al., 2019).

Besides, pharmacokinetic parameters showed that functional dyspepsia reduced the absorption of puerarin after oral administration of Jiawei-Xiaoyao-San (Qin et al., 2009). Compared with normal rats, the blood stasis rats exhibited a reduction in the AUC and C_max_ while there was an increase in the CL of puerarin after intravenous administration of total flavonoid from Gegen (Liu et al., 2013).

However, some pathological conditions played an opposite role on the absorption of puerarin, which could improve the bioavailability of puerarin. The puerarin concentration was significantly higher in the bacterial diarrhea rats than in normal rats (Ling et al., 2017). Puerarin in compound longmaining had a higher plasma concentration, slower elimination rate and higher bioavailability in myocardial ischemia rats (Dong et al., 2016).

Puerarin could be widely distributed in areas of the brain after administration. Brain diseases such cerebral vascular ischemia could influence the brain-targeting of puerarin (Li et al., 2017; Kong et al., 2019). More puerarin was transported into brain with a shorter t_1/2_ in middle cerebral artery occluasion rats model compared with control group via intranasal route (Li et al., 2017). After intraperitoneal injection, AUC_0-120 min_ of puerarin in the embolic hippocampus was significantly higher than that in the normal hippocampus at 40 and 20 mg/kg. C_max_ of puerarin in the embolic hippocampus was higher than that in the normal hippocampus at all doses (Kong et al., 2019).

### 2.2. Effects of single compound on pharmacokinetics of puerarin

Chinese medicines are often co-administered with other drugs or herbs in clinical practice. When drugs are co-administered to patients, drug-drug interactions should be considered carefully.

The absorption of puerarin is primarily mediated by P-gp and the multidrug resistance-associated protein (MRP) transporter in intestine. Co-administered compounds which are P-gp substrates could change the activity of P-gp, thus leading to drug-drug interactions, eventually affecting the pharmacokinetics of puerarin. Single compound such as verapamil (Chen et al., 2018; Zhou et al., 2018), cyclosporine A (Su et al., 2016), menthol (Zhang et al., 2016; 2017; Yang et al., 2018) and ligustrazine (Chen et al., 2018) could decrease the efflux ratio of puerarin. Its bioenhancement is mainly due to its inhibitory activity on P-gp mediated drug efflux. Besides the P-gp inhibitory effect, menthol also opened tight junction protein structure, and weaked the barrier capabilities of epithelial cells, eventually promoting the permeability of puerarin (Zhang et al., 2016; 2017). On the contrary, single compound such as glycyrrhizin (Zhao et al., 2018) and astragaloside IV (Liu et al., 2018) could increase the efflux ratio of puerarin through inducing P-gp activity. Compared with single administration, co-administration with gastrodin (Jiang et al., 2013) could improve the oral bioavailability of puerarin as well. However, their interaction mechanisms on pharmacokinetics remained unclear. The concurrent use of epalrestat (Sun et al., 2017) could slightly influence the pharmacokinetic profile of puerarin in rats, with no significant statistical difference.

Piperine is an active ingredient in white pepper. However, the pharmacokinetic profiles of puerarin were changed differently when coadministered with piperine and pepper (Liang et al., 2014). Upon concomitant oral administration with puerarin and piperine, as compared with the control group, the C_max_ and AUC_0-inf_ of puerarin was significantly improved, and T_max_ of puerarin was decreased gradually with the increase of the piperine dose, which was contrary to the concomitant oral administration with white pepper. The increased bioavailability of puerarin might be attributed to the inhibitory effect of piperine on intestinal CYP450 enzymes. The pharmacokinetic profiles of puerarin given by intravenous administration were changed by pretreatment with white pepper given orally, while it was not altered by piperine. The different drug-drug interactions with puerarin between piperine and white pepper indicated that besides piperine, other ingredients in white pepper might influence the pharmacokinetics of puerarin as well.

As mentioned above, single puerarin possessed the ability to across the blood-brain barrier, however to a relatively low degree. Some compounds could improve the absorption rate and distribution level of puerarin in the brain. Borneol and α-asarone could easily penetrate the blood–brain barrier and also promoted many other drugs into brain tissue (Gao et al., 2010; Yi et al., 2017; Wu et al., 2018). From the main brain distribution kinetic parameters, the values of t_1/2_ and AUC of the puerarin with borneol or α-asarone pretreatment group were obviously greater than that of the puerarin alone group, and the rates of distribution and elimination of the puerarin with borneol or α-asarone pretreatment group were obviously slower than those of the puerarin alone group, with statistically significant differences. Table 1 summarized the effects of single compound on pharmacokinetics of puerarin in rat plasma.

**Table 1:** The effects of single compound on pharmacokinetics of puerarin in rat plasma.

Compound	Administration	Control parameters	Modified parameters	References
verapamil	ig: puerarin(50 mg/kg); verapamil(100 mg/kg/day, 7 days)	C_max_:683.7 ± 51.2	C_max_:933.5 ± 75.8^a^	(Zhou et al., 2018)
		AUC_0–inf_:3687.3 ± 444.6	AUC_0–inf_:5006.1 ± 658.6^a^	
		t_1/2_:5.15 ± 0.46	t_1/2_:6.67 ± 0.51^a^	
		T_max_:0.51 ± 0.06	T_max_:0.34 ± 0.03^a^	
(+)-catechin	ig:puerarin(200 mg/kg); (+)-catechin(140 mg/kg)	C_max_:1070 ± 390	C_max_:4190 ± 2250^a^	(Su et al., 2016)
		AUC_0–12h_:3740 ± 1200	AUC_0–12h_:9290 ± 2360^b^	
		T_max_:0.49 ± 0.29	T_max_:0.47 ± 0.32	
	iv:puerarin(100 mg/kg); (+)-catechin(70 mg/kg)	C_max_:109610 ± 4340	C_max_:117330 ± 16610	(Su et al., 2016)
		AUC_0–12h_:91040 ± 11880	AUC_0–12h_:91660 ± 9610	
glycyrrhizin	ig: puerarin(50 mg/kg); glycyrrhizin(100 mg/kg/day, 7 days)	C_max_:761.25 ± 52.34	C_max_:456.32 ± 34.75^a^	(Zhao et al., 2018)
		AUC_0–inf_:4142.15 ± 558.51	AUC_0–inf_:2503.74 ± 447.57^a^	
		t_1/2_:4.37 ± 0.68	t_1/2_:5.45 ± 0.52	
		T_max_:0.51 ± 0.05	T_max_:0.35 ± 0.02	
		CL:12.20 ± 1.53	CL:20.47 ± 3.25^a^	
astragaloside IV	ig: puerarin(50 mg/kg); astragaloside IV (100 mg/kg/day, 7 days)	C_max_:760 ± 36.9	C_max_:467 ± 29.8^a^	(Liu et al., 2018)
		AUC_0–inf_:4097 ± 625	AUC_0–inf_:2330 ± 761^a^	
		t_1/2_:5.18 ± 0.54	t_1/2_:4.65 ± 0.36	
		T_max_:0.49 ± 0.07	T_max_:0.36 ± 0.05^a^	
		CL:11.9 ± 1.38	CL:22.4 ± 2.97^a^	
gastrodin	ig: puerarin(400 mg/kg); gastrodin(40 mg/kg)	C_max_:490 ± 150	C_max_:2010 ± 380^b^	(Jiang et al., 2013)
		AUC_0–inf_:1160 ± 320	AUC_0–inf_:12400 ± 1510^b^	
		t_1/2_:1.33 ± 0.350	t_1/2_:4.00 ± 1.16^b^	
		T_max_:1.95 ± 1.15	T_max_:0.57 ± 0.34^a^	
		CL:365 ± 100	CL:32.7 ± 4.47^b^	
	iv: puerarin(20 mg/kg); gastrodin(20 mg/kg)	C_max_:71400 ± 6500	C_max_:65800 ± 9730	
		AUC_0–inf_:21200 ± 1830	AUC_0–inf_:24100 ± 3880	
		t_1/2_:1.44 ± 0.99	t_1/2_:2.22 ± 0.78	
		CL:0.947 ± 0.0872	CL:0.850 ± 0.148	
epalrestat	iv: puerarin(30 mg/kg) ig: epalrestat(15 mg/kg)	C_max_:3800.6 ± 1209.7	C_max_:4542.5 ± 1776.5	(Sun et al., 2017)
		AUC_0–inf_:6104.0 ± 1269.5	AUC_0–inf_:6860.5 ± 3239.6	
		t_1/2_:1.1 ± 0.1	t_1/2_:1.6 ± 0.7	
piperine	ig: puerarin(400 mg/kg); piperine(10 mg/kg)	C_max_:18661 ± 5080	C_max_:24286 ± 5662^a^	(Liang et al., 2014)
		AUC_0–inf_:94203 ± 17015	AUC_0–inf_:125689 ± 20794^a^	
		t_1/2_:4.286 ± 0.871	t_1/2_:4.703 ± 1.738	
		T_max_:0.428 ± 0.203	T_max_:0.413 ± 0.272	
		CL:4.335 ± 1.217	CL:3.439 ± 1.158	
	ig: puerarin(400 mg/kg); piperine(20 mg/kg)		C_max_:30629 ± 8636^b^	
			AUC_0–inf_:148211 ± 26853^b^	
			t_1/2_:5.052 ± 1.797	
			T_max_:0.385 ± 0.313	
			CL:2.525 ± 1.302^a^	
	iv: puerarin(40 mg/kg); piperine(10 mg/kg)	AUC_0–inf_:90221 ± 17419	AUC_0–inf_:98366 ± 23219	(Liang et al., 2014)
		t_1/2_:0.715 ± 0.201	t_1/2_:0.702 ± 0.204	
		CL:0.433 ± 0.109	CL:0.389 ± 0.118	
	iv: puerarin(40 mg/kg); piperine(20 mg/kg)		AUC_0–inf_:91982 ± 12127	
			t_1/2_:0.652 ± 0.247	
			CL:0.441 ± 0.058	
edaravone	iv: puerarin(62.5 mg/kg); edaravone(3.75 mg/kg)	AUC:13800 ± 1710	AUC:15380 ± 1590	(Gao et al., 2010)
		t_1/2_:0.05 ± 0.02	CL:4.10 ± 0.42	
		CL:4.59 ± 0.58	t_1/2_:0.22 ± 0.03^b^	
borneol	ig: puerarin(200 mg/kg); borneol(25 mg/kg)	C_max_:1076.02 ± 160.52	C_max_:1264.67 ± 121.95	(Yi et al., 2017)
		AUC_0–12h_:3511.42 ± 583.86	AUC_0–12h_:3877.15 ± 367.46	
		t_1/2_:1.90 ± 0.32	t_1/2_:2.55 ± 0.65	
		T_max_:1.00 ± 0.00	T_max_:0.69 ± 0.24^a^	
	ig: puerarin(200 mg/kg); borneol(50 mg/kg)		C_max_:1645.25 ± 193.03^b^	
			AUC_0–12h_:6788.15 ± 1288.18^b^	
			t_1/2_:2.76 ± 0.27^b^	
			T_max_:0.60 ± 0.14^b^	
	ig: puerarin(200 mg/kg); borneol(100 mg/kg)		C_max_:2108.22 ± 140.54^b^	
			AUC_0–12h_:7594.67 ± 649.29^b^	
			t_1/2_:1.93 ± 0.41	
			T_max_:1.00 ± 0.00	
α-asarone	ig: puerarin(20 mg/kg); α-asarone (25 mg/kg)	Brain:	Brain:	(Wu et al., 2018)
		C_max_:13.24 ± 4.28	C_max_:35.16 ± 12.442^b^	
		AUC_0–12h_:48.05 ± 8.54	AUC_0–12h_:86.02 ± 7.927^b^	
		t_1/2_:11.33 ± 4.07	t_1/2_:12.81 ± 5.64	
		T_max_:0.31 ± 0.13	T_max_:0.61 ± 0.52	
		Plasma:	Plasma:	
		C_max_:439.15 ± 137.02	C_max_:368.58 ± 260.71	
		AUC_0–12h_:938.29 ± 208.57	AUC_0–12h_:1003.32 ± 185.60	
		t_1/2_:1.66 ± 0.59	t_1/2_:3.39 ± 0.824^a^	
		T_max_:0.38 ± 0.22	T_max_:0.85 ± 0.61	

iv: intravenous administration; ig: intragastrically administration.

C_max_(μg/L): maximum concentration; CL(L/h/kg): apparent clearance; AUC_0–t_(μg·h/L): area under concentration-time curve from time 0 to last observed time; AUC_0–inf_(μg·h/L): area under concentration-time curve from time 0 to infinity; t_1/2_(h): half-life; T_max_(h): time to reach maximum concentration.

^a^*p* < .05 versus control group given puerarin only; ^b^*p* < .01 versus control group given puerarin only.

On the contrary, puerarin could affect the pharmacokinetics of some active compounds which were substrates of P-gp, MRP and CYP 450. Puerarin could significantly change the pharmacokinetic profiles of triptolide in rats probably though inhibiting the activity of P-gp or CYP3A4 (Wang et al., 2019). CYP3A4 enzyme was also involved in the metabolism of ivabradine, the AUC and C_max_ of ivabradine in rats that were pretreated with puerarin were significantly higher than that of the ivabradine control group (Zhang et al., 2016). The CYPs involved in warfarin metabolism include CYP1A1, 2C9, 2C19 and 2B1 in humans. The induction of rCyp2b1, 2c6 and 1a1 after administration of puerarin altered the metabolism of warfarin, intravenously administered puerarin altered the pharmacokinetics of warfarin significantly by shortening t_1/2_, decreasing AUC_0–96h_ and increasing the clearance of warfarin (Ge et al., 2017).

### 2.3. Effects of herbs on pharmacokinetics of puerarin

Compatible‘herb-pairs’in traditional Chinese medicine play an important role in oriental traditional medicine. Herbal preparations such as Gegenqinlian decoction, compound Longmaining are composed of Gegen and other compatible Chinese medicines. The compatible herbs in the prescriptions could influence the pharmacokinetics of puerarin. The pharmacokinetic parameters of puerarin from Gegenqinlian decoction were absorbed more effectively with slow elimination in rat plasma than from *Radix Puerariae* extracts (Zhang et al., 2014).

Besides Gegen, Gegenqinlian decoction are composed of *Scutellaria baicalensis* (rich in baicalin), *Coptis chinensis* (rich in berberine) and *Glycyrrhiza uralensis* (rich in glycyrrhizic acid). Different proportions of baicalin, glycyrrhizic acid and berberine had certain influence on intestinal permeability of puerarin. Glycyrrhizic acid could significantly inhibit the intestinal absorption of puerarin, while high concentrations of baicalin and berberine could promote the absorption of puerarin (Zhang et al., 2012; Liu et al., 2014).

Bai et al. (2017) investigated the effects of compatible herbs in compound Longmaining on the intestinal absorption of puerarin. In the whole intestine of rats, compound Longmaining could significantly promote the absorption of puerarin. In the duodenum and ileum, *Dioscoreae nipponicae rhizoma* played a significant role in promoting absorption of puerarin. In jejunum and colon, *Dioscoreae nipponicae rhizoma* and *Chuanxiong rhizoma* have a synergistic effect in promoting absorption of puerarin.

The intestinal absorption of puerarin could be improved by *Gastrodiae rhizome* extracts (Liu et al., 2019), *Angelica dahurica* extracts (Liang et al., 2012), *Radix angelicae dahuricae* extracts (Liang et al., 2012; Liao et al., 2014) and *Chuanxiong rhizome* extracts (Chen, 2018). *Chuanxiong rhizoma* enhanced the absorption and pharmacokinetics of puerarin through elevating puerarin solubility, regulating P-glycoprotein efflux and reducing claudin-5 expression. However, due to the complex compatible compositions of traditional Chinese medicine, most of the herb-pairs interaction mechanisms remained unknown.

## 3. Drug delivery systems of puerarin

### 3.1. Microemulsions and self-microemulsifying drug delivery systems

Microemulsions, as promising drug delivery systems, have been proved to be effective in enhancing the bioavailability of poorly water-soluble drugs. Microemulsions are isotropic, thermodynamically stable transparent systems consisting of an oil phase, a water phase, and a surfactant, often in combination with a co-surfactant. Microemulsions are structurally divided into oil-in-water, water-in-oil, and bicontinuous microemulsions (Callender et al., 2017).

Upon oral administration, puerarin-loaded microemulsions significantly improved the bioavailability of puerarin from 1.2-fold to 15.2-fold compared with corresponding puerarin control groups (Wu et al., 2009; Yu et al., 2011; Tang et al., 2013; Liao et al., 2015; Wu et al., 2018). Powerful absorption enhancing agents such as ethyl oleate (Yu et al., 2011; Tang et al., 2013) and N-trimethyl chitosan chloride (Liao et al., 2015) were added into the formulation, not only as the oil phase, but also improved the oral bioavailability of puerarin. When N-trimethyl chitosan chloride was added to the microemulsion formulation, the relative bioavailability was enhanced more than five times compared with control microemulsion (Liao et al., 2015). Through combining phospholipid complex technology and microemulsion, the relative oral bioavailability of puerarin phospholipid complex was 3.16-fold higher than puerarin(Wu et al., 2018).

The remarkable oral bioavailability promotion effects of microemulsions were attributed to many aspects. Firstly, microemulsions could improve the solubility of puerarin. The solubility of puerarin in microemulsion was significantly improved from 4.58 mg/ml to 27.8 mg/ml compared of crude puerarin in water (Yu et al., 2011). Furthermore, lymphatic transport was a major contributor to intestinal absorption of puerarin and subsequently to its oral bioavailability (Wu et al., 2011; Tang et al., 2013). More oil in the microemulsion formulation resulted in more lymphatic transport of puerarin (Tang et al., 2013). Besides oral administration, nasal (Yu et al., 2011) and intravenous (Yue et al., 2008) administration of puerarin microemulsion could improve the absolute bioavailability as well. Table 2 summarized the compositions and achieved improvements of microemulsions on puerarin.

**Table 2.** Compositions and achieved improvements of microemulsion on puerarin.

Compositions (%,w/w)	Droplet size	Puerarin content	Improvements	References
	Mean(nm)	(mg/ml)		
ethyl oleate: cremophor rh40: propylene glycol: water(2.1:12.6:6.3:79.0)	13.50 ± 0.58	11.32 ± 0.16	Significantly improved bioavailability compared with suspension following oral administration (1.84-fold and 1.95-fold higher, respectively)	(Tang et al., 2013)
ethyl oleate: cremophor rh40: propylene glycol: water(35.7:26.9:26.7:10.7)	24.04 ± 1.02	23.14 ± 0.32		
ethyl oleate: tween 80: glycerin: water(1.6:20:20:58.4)	23.4 ± 2.2	27.8 ± 2.4	Rapid absorption and high bioavailability (34.58%) after nasal administration, compared with puerarin suspension (4.13%) and puerarin-loaded microemulsion (13.54%) after oral administration	(Yu et al., 2011)
soybean oil: soybean lecithin: ethyl	40.2 ± 5.9	NG	AUC was 15.82-fold higher (37.91% vs 2.5%), MRT increased 5.289 times than puerarin suspension upon oral administration	(Wu et al., 2009)
lactate: water(38:22:22:18)				
lecithin: anhydrous ethanol:	21.93	49.42	Relative oral bioavailability was 3.16-fold higher than puerarin	(Wu et al., 2018)
LABRAFIL®M1944CS: water (18.18:36.36:				
36.36:9.10)				
soybean oil(12 g); egg lecithin(1.2 g); synperonic F68(0.1 g); glycerol(2.5 g); α-tocopherol(300 mg); water(90.0 g)	188.14	10	After iv administration, AUC was 1.718-fold higher, CL significantly decreased, t_1/2_ and MRT increased, compared with puerarin group	(Yue et al., 2008)

NG: not given.

Self-emulsifying drug delivery systems(SMEDDS) are isotropic mixture of oils, surfactants, and cosurfactants, which are emulsified in aqueous media under conditions of gentle agitation. Compared with microemulsions, one of the greatest advantages of SMEDDS is their spontaneous emulsification and formation of an emulsion, microemulsion or nanoemulsion in aqueous media (Cerpnjak et al., 2013). Quan et al. (2007) investigated an optimized SMEDDS formulation which consisted of oil (17.5%), Tween-80 (34.5%) and propylene glycol (34.5%), its absolute bioavailability in beagle dogs after oral administration was about 24.8%. To overcome the problems of low stability of liquid SMEDDS, solid SMEDDS was prepared by spherical crystallizaztion(Cheng et al., 2016), there was no significant difference of the bioavailability between the liquid SMEDDS and solid SMEDDS, while the solid SMEDDS could better control the release of the drug compared with the liquid SMEDDS. Zhang et al. (2012) prepared a SMEDDS sustained-release pellet for oral puerarin delivery via extrusion-spheronization. The absolute bioavailability of puerarin-SMEDDS sustained-release pellets was enhanced by approximately 2.6-fold compared with puerarin tablet.

### 3.2. Dendrimers

Polyamidoamine(PAMAM) dendrimers are the most extensively investigated dendrimers, which have been widely used in drug delivery systems. PAMAM dendrimers are water-soluble molecules, which possess abilities to enhance solubility, stability and oral bioavailability of various drugs (Chauhan, 2018). Gu et al. (2013) investigated the effect of PAMAM dendrimers on promoting the solubility and oral bioavailability of puerarin. The water solubility of puerarin was significantly improved by full generation dendrimers with amine terminated surface functional groups, while was no significantly improved by half generation dendrimers with carboxylate surface functional groups. The solubilization was primarily due to the electrostatic interactions between the primary amine groups of the PAMAM dendrimer and the phenolic hydroxyl groups of puerarin. The pharmacokinetics parameters T_max_, C_max_, and AUC_0–8h_ of puerarin suspension solution and puerarin–G2 dendrimer complex solution were 0.76 h, 1.50 µg/mL, 7.33 µg·h/mL and 0.33 h, 6.49 µg/mL, 14.02 µg·h/mL, respectively.

PAMAM dendrimers were successfully applied in ocular drug delivery system of puerarin (Yao et al., 2010; 2010; Wang et al., 2011; Yao et al., 2011). Following ocular administration on rabbits, the AUC_0-inf_, C_max_ and t_1/2_ values of puerarin in PAMAM dendrimer complex was significantly higher than those in the control group, and which was related to the generation of PAMAM dendrimer (Wang et al., 2011; Yao et al., 2011). After cornea was treated with PAMAM dendrimers, the permeability coefficient of puerarin was enhanced by 2.48 (G3), 1.99 (G4) and 1.36 (G5) times on average, respectively. The permeability coefficient of puerarin was linear correlated to the generation of PAMAM dendrimer, higher generation of PAMAM dendrimer interact with cornea or loosen the epithelium cell junctions more easily than lower generation to increase the flux of puerarin (Yao et al., 2010). PAMAM dendrimer slowed the release rate of puerarin in cornea compared with puerarin eye drops, which could be explained by intermolecular hydrogen-bonding interactions between puerarin and PAMAM dendrimers. Besides, the in vitro release rate of puerarin with full generation PAMAM dendrimers was lower than that with half generation dendrimers (Yao et al., 2010).

### 3.3. Nanoparticles

Nanoparticles as drug carriers have attracted widespread attention due to the special medical value. Drugs can be embedded or dissolved in this system, with the solubility of poorly hydrophilic drugs improving. In addition, nanoparticles also possess the ability to improve the targeted effect and reduce the toxicity and side effects. Solid lipid nanoparticles and polymer nanoparticles are the focus of attention in clinical researches (Li et al., 2018).

Several puerarin-loaded solid lipid nanoparticles were prepared in recent years. Luo et al. (2011, 2013) used the solvent injection method to prepare solid lipid nanoparticles for oral administration of puerarin. Compared with puerarin suspension, puerarin solid lipid nanoparticles were rapidly absorbed, as evidenced with a shorter T_max_, the relative bioavailability of puerarin improved more than three times. Following administration of the puerarin solid lipid nanoparticles, tissue concentrations of puerarin increased especially in the target organs such as the heart and brain. Similar metabolic profiles were found in rat plasma and urine, which indicated incorporation of puerarin into solid lipid nanoparticles didn’t change the metabolic pathway. Dong et al. (2017) prepared RGD modified and PEGylated solid lipid nanoparticles loaded with puerarin by the solvent evaporation method. After intravenous administration, the bioavailability of puerarin nanoparticles was significantly increased, AUC increased from 52.93 for free puerarin to 176.5 (mg· h/mL) for puerarin nanoparticles, T_1/2_ increased from 0.73 h for free PUE to 2.62 h for puerarin nanoparticles, and higher drug concentration in heart and plasma was found compared with other puerarin formulations.

Puerarin nanoparticles loaded with polymers such as PEGylated mesoporous silica (Liu et al., 2016), aliginate-chitosan (Hou et al., 2014), poly(butylcyanoacrylate) (Zhao et al., 2013) and HP-β-CD- poly(D,L-lactic-co-glycolic acid) (Tao et al., 2013) were prepared to improve the bioavailability of puerarin. The releases of puerarin from PEGylated mesoporous silica and aliginate-chitosan nanoparticles were influenced by the pH of the test medium, with release rate much faster at lower pH than at higher pH. PEGylated mesoporous silica nanoparticles improved blood compatibility over the mesoporous silica nanoparticles and reduced hemolysis induced by puerarin. Puerarin-loaded poly(butylcyanoacrylate) and HP-β-CD-poly (D, L-lactic-co-glycolic acid) nanoparticles both increased and prolonged the puerarin concentration in the brain. A puerarin nanoparticle was synthesized based on glycyrrhetinic acid-PEG-PBLA, which could obviously increase the water solubility, bioactivity and litter half-life of puerarin and heighten the liver-targeted drug delivery (Xiao et al., 2018). Zhang et al. (2016) prepared puerarin nanoparticles by an emulsion solvent evaporation method, followed by freeze-drying. The oral bioavailability of puerarin nanoparticles was 2.83 times of raw puerarin.

### 3.4. Nanocrystals

Nanocrystal technology is proven to be effective to improve the bioavailability of poorly soluble drugs in animals via different administration routes by many times (Gao et al., 2012). Wang et al. (2012) first prepared puerarin nanocrystals in 2012, the developed formulations were intravenous administered to beagle dogs. Puerarin dissolution velocity and saturation solubility were enhanced by the nanocrystals, while the C_max_ and clearance was significantly reduced compared to the puerarin solution. However, later researches on puerarin nanocrystals showed that compared with puerarin control group, both the C_max_ and AUC of puerarin nanocrystals were enhanced, the absolute bioavailability was increased 4.47-fold (Yi et al., 2015) and 7.6-fold (Tu et al., 2013) respectively.

The combinations of various preparation technologies should be paid attention to. Zhang et al. (2018) prepared an oral drug nanocrystals self-stabilized Pickering emulsion, the relative bioavailability of puerarin nanocrystal emulsion was significantly higher than puerarin coarse power suspension, nanocrystal suspension, and surfactant emulsion. The prepared puerarin nanocrystals were successfully applied to improve brain accumulation for the treatment of Parkinson's disease (Xiong et al., 2019). Puerarin nanocrystals attenuated dopamine depletion, ameliorated 1-methyl-4-phenyl-1,2,3,6-tetrahydropyridine-induced behavioral deficits, and enhanced the levels of dopamine and its metabolites.

### 3.5. Others

To improve transcorneal permeation, prolong residence time in the eye and alleviate some respiratory and gastrointestinal side effects, various vehicles (Qi et al., 2007; Wu et al., 2007; Xu et al., 2010; Liu et al., 2011; Yu et al., 2012) were prepared in ophthalmic delivery of puerarin. Gelucire44/14 (Liu et al., 2011) and sperminiated pullulans (Yu et al., 2012) as effective absorption enhancers, promoted ocular bioavailability of puerarin. Polymer in situ gelling vehicles performed better in retaining puerarin in eye, enhanced ocular bioavailability and patience compliance than puerarin eye drops did (Qi et al., 2007; Wu et al., 2007). A soft contact lens vehicle composed of Poly (2-hydroxy-ethyl methacrylate-co-N-vinylpyrrolidone-co-methyl acrylate) was prepared (Xu et al., 2010), which had a remarkable loading capacity and extended the mean resident time of puerarin.

Xie et al. (2013) used mechanochemical technology to prepare puerarin-2-hydroxypropyl-β-cyclodextrin inclusion complex. In comparison to puerarin, the solubility of the inclusion complex was 25.33-fold higher and the drug release amount reached 79.44% at 15 min, 2.76-fold higher. A novel puerarin gastric floating system was prepared by a 3 D extrusion-based printing technique, with good gastric residence time and controlled release ability (Li et al., 2019). In addition, other carriers used for improving the bioavailability of puerarin such as phospholipid complex (Li et al., 2006), microsphere (Song et al., 2016), micelle (Li et al., 2018), liposome (Deng et al., 2010; Zhao et al., 2016) and spray (Lu et al., 2007) were reported.

## 4. Conclusion

We concluded the pharmacokinetics of puerarin in different pathological conditions. Diseases originated intestinal gene and protein overexpression of Ugt1a1, Ugt1a7 and P-gp played important roles in the pharmacokinetics of puerarin. The drug-drug interactions should be taken seriously when co-administrated. However, besides regulating the activities of P-gp and the MRP transporter in intestine, sufficient basic knowledge about their action mechanisms are needed, especially in compound prescriptions of traditional Chinese medicine.

Nanotechnologies have been successfully developed in the past several decades, it’s still a relatively new field to develop drug delivery systems for poor bioavailability drugs due to their limited solubility and absorption. Various nanotechnologies and preparation technologies such as microemulsions and SMEDDS, dendrimers, nanoparticles and nanocrystals were applied to improve the bioavailability of puerarin. Considering the administration route, PAMAM dendrimer is promising to serve as a corneal permeation enhancer in ophthalmic drug delivery system, possessing the ability to increase puerarin solubility and corneal residence time. lipid-based drug delivery systems such as microemulsions and SMEDDS show great advantages to improve the solubility and bioavailability of poorly water-soluble drugs. The choice of surfactants is limited since very few are acceptable for oral administration. Safety is a major determining factor when choosing a surfactant, the nonionic surfactants are the most widely recommended and used ones (Cerpnjak et al., 2013). Nanoparticles comprise of a multitude of various manufactured materials, although several nanoparticles have been approved, some nanoparticles (such as metallic and carbon-based particles) tend to display toxicity (Wolfram et al., 2015). Due to the complexity of compositions, more effective quality evaluations of nanocarriers are required in order to achieve biocompatibility and desired activity. In addition, the safety of nanocarriers in clinical applications need in-depth research.
